# Endpoint resuscitation-based prediction model for early mortality of severe sepsis and septic shock

**DOI:** 10.1186/cc13252

**Published:** 2014-03-17

**Authors:** R Sinto, S Suwarto, R Sedono, K Harimurti, A Sejati

**Affiliations:** 1 Faculty of Medicine, University of Indonesia, Jakarta, Indonesia

## Introduction

There is an unknown role for macrocirculation and microcirculation endpoint resuscitation, which are combined as the component of a prediction model for early mortality of patients with severe sepsis and septic shock [1][2]. The aim of this study is to develop a prediction model for early mortality (first 120 hours after onset [3]) of patients with severe sepsis and septic shock based on macrocirculation and microcirculation endpoint resuscitation.

## Methods

A retrospective cohort study was conducted in adult patients with severe sepsis and septic shock hospitalized in the ICU, Cipto Mangunkusumo Hospital, Indonesia. Patients' outcome and time to outcome were observed during the first 120 hours after severe sepsis and/or septic shock onset. Independent predictors for early mortality were identified by Cox's proportional hazard regression analysis and each was quantified to develop an early mortality prediction model. The calibration and discrimination abilities of the model were determined.

## Results

Subjects consisted of 268 patients. Early mortality developed in 70 patients. Two independent predictors for early mortality were: lactate clearance <10% (adjusted hazard ratio (HR) 11.81 (95% CI 6.50 to 21.46)) and number of organ dysfunctions (two organs, adjusted HR 1.47 (95% CI 0.58 to 3.72); >2 organs, adjusted HR 3.79 (95% CI 1.65 to 8.69)). A scoring system as the predictive model was performed by assigning 1 point for two organ dysfunctions, 6.5 points for >2 organ dysfunctions, and 12 points for lactate clearance <10%. This scoring system was stratified into two levels: low-risk (score <12, probability for early mortality 7.8%) and high-risk (score ≥12, probability for early mortality 72.3%) groups. The Hosmer-Lemeshow test revealed good precision (*P *= 0.745) and the area under the receiver operating characteristic curve showed very good discrimination ability (0.91 (95% CI 0.87 to 0.97)).

## Conclusion

A prediction model for early mortality of patients with severe sepsis and septic shock can be developed based on two parameters, lactate clearance and number of organ dysfunctions. A model has been developed to predict and classify mortality risk.
